# Are there shared neural correlates between dyslexia and ADHD? A meta-analysis of voxel-based morphometry studies

**DOI:** 10.1186/s11689-019-9287-8

**Published:** 2019-11-21

**Authors:** Lauren M. McGrath, Catherine J. Stoodley

**Affiliations:** 1 Department of Psychology, University of Denver, Frontier Hall, 2155 S. Race St., Denver, CO 80208 USA; 2 Department of Psychology and Center for Behavioral Neuroscience, American University, Washington, DC USA

**Keywords:** Voxel-based morphometry, Meta-analysis, Dyslexia, Attention-deficit/hyperactivity disorder, Caudate

## Abstract

**Background:**

Dyslexia and Attention-deficit/hyperactivity disorder (ADHD) are highly comorbid neurodevelopmental disorders (estimates of 25–40% bidirectional comorbidity). Previous work has identified strong genetic and cognitive overlap between the disorders, but neural overlap is relatively unexplored. This study is a systematic meta-analysis of existing voxel-based morphometry studies to determine whether there is any overlap in the gray matter correlates of both disorders.

**Methods:**

We conducted anatomic likelihood estimate (ALE) meta-analyses of voxel-based morphometry studies in which individuals with dyslexia (15 studies; 417 cases, 416 controls) or ADHD (22 studies; 898 cases, 763 controls) were compared to typically developing controls. We generated ALE maps for dyslexia vs. controls and ADHD vs. controls using more conservative (*p* < .001, *k* = 50) and more lenient (*p* < .005, *k* = 50) thresholds. To determine the overlap of gray matter correlates of dyslexia and ADHD, we examined the statistical conjunction between the ALE maps for dyslexia vs. controls and ADHD vs. controls (false discovery rate [FDR] *p* < .05, *k* = 50, 5000 permutations).

**Results:**

Results showed largely distinct gray matter differences associated with dyslexia and ADHD. There was no evidence of statistically significant gray matter overlap at our conservative threshold, and only one region of overlap in the right caudate at our more lenient threshold. Reduced gray matter in the right caudate may be relevant to shared cognitive correlates in executive functioning and/or procedural learning. The more general finding of largely distinct regional differences in gray matter between dyslexia and ADHD suggests that other neuroimaging modalities may be more sensitive to overlapping neural correlates, and that current neuroimaging recruitment approaches may be hindering progress toward uncovering neural systems associated with comorbidity.

**Conclusions:**

The current study is the first to meta-analyze overlap between gray matter differences in dyslexia and ADHD, which is a critical step toward constructing a multi-level understanding of this comorbidity that spans the genetic, neural, and cognitive levels of analysis.

## Background

Dyslexia (also known as DSM-5 Specific Learning Disorder with Impairment in Reading) and Attention-deficit/hyperactivity disorder (ADHD) are both prevalent developmental disorders (5–10%) with a high, bidirectional comorbidity rate (25–40%) [1, 2]. One theoretical advancement that is guiding the study of comorbidity is the shift from single deficit to multiple deficit models in developmental neuropsychology [3]. The multiple deficit model stipulates that there are multiple, probabilistic predictors of developmental disorders across levels of analysis and that comorbidity arises because of risk factors that are shared by disorders [3]. This multiple deficit framework has been useful for advancing the science of comorbidity, particularly for integrating the genetic, neural, and cognitive levels of analysis to explain comorbidity. There is strong evidence for shared genetic and neuropsychological risk factors that contribute to the dyslexia-ADHD comorbidity; what is missing are the potential overlapping neural risk factors that can connect these levels of analysis. This gap at the neural level is preventing the specification of a fully integrated model of the dyslexia-ADHD comorbidity that spans multiple levels of analysis.

At the genetic level of analysis, the bulk of the evidence supports the correlated liabilities model of comorbidity between dyslexia and ADHD [4], which posits that shared genetic influences cause both disorders to manifest in the same child more often than expected by chance. Evidence in support of the correlated liabilities model is derived from multivariate behavioral genetic studies of twins, which can establish the extent to which genetic influences on one disorder overlap with genetic influences on the second disorder [5]. One way to quantify the extent of the genetic overlap is with a statistic called the genetic correlation, which ranges from 0 (genetic influences on one trait are not associated with the second trait) to 1 (all of the genetic influences on one trait also influence the second trait) [5]. One way to interpret the genetic correlation is that it expresses the probability that a gene associated with one trait will also be associated with the second trait [6]. Estimates of the genetic correlation between dyslexia and ADHD are quite strong, in the range of .50 and extending up to .70 in some studies [7].

At the neuropsychological level of analysis, there is also evidence for shared risk factors, most notably deficits in processing speed [8–18] and aspects of executive functioning, including working memory [17, 19–24], inhibition [17, 25, 26], and sustained attention [17, 26].

In comparison to the progress in understanding the comorbidity of dyslexia and ADHD at the genetic and neuropsychological levels of analysis, there is a striking gap at the neural level of analysis. For example, there are only a handful of structural neuroimaging studies that have directly examined the comorbid dyslexia+ADHD group [27–31]. The bulk of neuroimaging designs either (a) recruit “pure” groups without comorbidities or (b) compare separate groups based on comorbidity status (i.e., dyslexia, ADHD, dyslexia+ADHD). While both of these strategies are useful for specific research questions, neither directly addresses why the disorders co-occur in the first place. In fact, both designs address the question of what *distinguishes* one disorder from another, rather than identifying transdiagnostic regions where they have shared features.

Such a transdiagnostic approach has been rare in developmental neuroimaging samples to date (for exceptions see [32, 33]), but there is a notable meta-analytic study in the adult psychiatric neuroimaging literature that can provide a guiding framework. Goodkind et al. [34] analyzed structural neuroimaging studies of clinical disorders vs. controls. The clinical disorders covered a broad range (i.e., schizophrenia, bipolar disorder, major depressive disorder, substance use disorders, obsessive-compulsive disorders, and anxiety disorders). The authors meta-analyzed the existing voxel-based morphometry (VBM) studies of each disorder and then conducted a conjunction analysis to identify regions that were common across disorders. Results pointed to the dorsal anterior cingulate cortex and the bilateral insula as regions with less gray matter across clinical disorders compared to controls. Both of these regions have been associated with executive dysfunction, which is consistent with cognitive studies reporting that executive dysfunction is often a cross-cutting cognitive phenotype across a diverse range of psychiatric and neurodevelopmental disorders [34–37], including dyslexia and ADHD. More generally, these findings illustrate the potential to identify transdiagnostic correlates even in samples that were not initially recruited to directly study comorbidity.

In the dyslexia and ADHD literature, there is one meta-analysis completed by one of the authors (CJS) that directly tested for brain regions associated with both dyslexia and ADHD, but it focused exclusively on the cerebellum [38]. This study was a meta-analysis of cerebellar VBM studies in dyslexia and ADHD. There was no overlap between cerebellar clusters associated with dyslexia and ADHD, but there was potential functional overlap in the ventral attention system because clusters identified in the cerebellum for both disorders were implicated in this attentional network [38].

Given the sparse literature on shared neural correlates between dyslexia and ADHD, it is useful to speculate about neural systems that might be implicated in both disorders. For dyslexia, the most commonly implicated neural correlates involve a reading network that comprises left occipitotemporal regions, left temporoparietal regions, and the left inferior frontal gyrus [39]. In ADHD, the most frequently implicated regions include the prefrontal cortex and striatum [40–42]. While there are not obvious points of overlap in the canonical regions implicated in both disorders, it remains possible that there are regions of overlap that have received less attention because they are not part of these canonical regions.

As a result, in the current study, we utilize a quantitative meta-analytic approach to systematically test for common neural correlates. Specifically, we examine differences in gray matter volume identified via voxel-based morphometry (VBM) methods [43, 44]. VBM is the most widely-used automated technique for the analysis of structural brain images. While differences in functional activation and structural and functional connectivity are also implicated in dyslexia and ADHD, we chose to focus on gray matter correlates for this initial study because the VBM literature is robust in both dyslexia and ADHD (*N* = 15 dyslexia studies, *N* = 22 ADHD studies). The meta-analytic approach allows us to be inclusive of studies across the lifespan in order to maximize sample size, while also examining heterogeneity across age. Importantly, our analytic strategy is designed to identify transdiagnostic gray matter correlates as compared to the prevailing neuroimaging designs, which focus on distinctions between the disorders. The overall goal of this meta-analysis is to identify overlap in brain regions associated with dyslexia or ADHD in VBM studies of these disorders. Such areas of overlap will advance our understanding of the dyslexia/ADHD comorbidity at the neural level, which is a critical gap in the literature given important advances at both the etiological and neuropsychological levels of analysis in understanding this comorbidity.

## Methods

In reporting the results of this systematic meta-analysis, we have followed the guidelines proposed by Müller et al. [45] for reporting neuroimaging meta-analyses, which are aligned with recommendations from PRISMA (Preferred Reporting Items for Systematic Reviews and Meta-analyses) [46] (see Checklist in Additional file 1: Table S1).

### Literature search

Pubmed (http://www.ncbi.nlm.nih.gov/pubmed) was used as a primary search database with follow-up searches completed using Google scholar (https://scholar.google.com/). The literature search was completed in April 2018. For the PubMed searches, we used curated medical subject headings for dyslexia (“dyslexia”) and ADHD (“Attention Deficit Disorder with Hyperactivity”) as well as permutations of relevant keywords (e.g., dyslexia, reading disability, reading disorder, ADHD, attention-deficit). To narrow the vast neuroimaging literatures to those studies using VBM methods, we used permutations of the phrases “voxel-based” and “gray matter.” The VBM method was first published in 2000, so we limited our search to publications between 1 January 1999 and 30 April 2018. The PubMed search syntax for dyslexia was as follows: (Dyslexia [MeSH] OR dyslex* OR reading disab* OR reading disorder*) AND (“voxel-based” OR “voxel based” OR VBM OR “gray matter” OR “grey matter”) AND (“1999/01/01”[Date - Publication]“2018/04/30”[Date - Publication]) AND English[Language]. The PubMed search syntax for ADHD was as follows: (Attention Deficit Disorder with Hyperactivity [MeSH] OR ADHD OR attention*deficit) AND (“voxel-based” OR “voxel based” OR VBM OR “gray matter” OR “gray matter”) AND (“1999/01/01”[Date - Publication]: *“2018/04/30”[Date - Publication]) AND English[Language].* To ensure that we had identified all relevant studies, we also cross-referenced our searches with previous VBM meta-analyses for dyslexia [47–49] and ADHD [﻿32﻿, 40, 50, 51]. Additional searches with the same keywords in Google scholar did not turn up additional papers that met inclusion criteria beyond those identified through PubMed and existing meta-analyses.

**Fig. 1:**
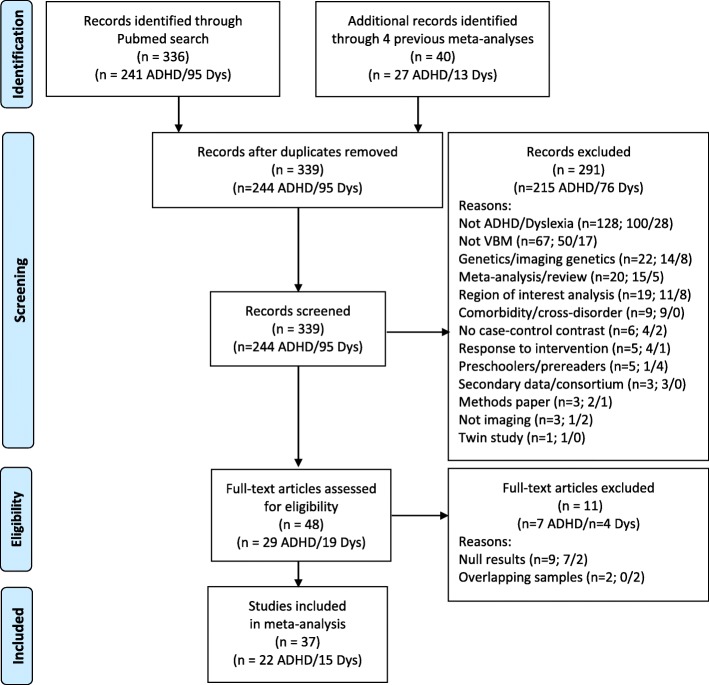
PRISMA flow chart of literature search and screening for ADHD and dyslexia voxel-based morphometry case-control studies. From [46]. For more information, visit www.prisma-statement.org

In order to be included, studies were required to use whole-brain voxel-based morphometry (VBM) methods and to compare the clinical group with typically developing age-matched comparison groups. Methodological exclusion criteria included studies that reported non-VBM or only region-of-interest analyses of structural MRI data, studies in which results were not reported in standard coordinate space (Montreal Neurological Institute [MNI] [53] or Talairach and Tournoux [54]), studies reporting incomplete coverage of the whole brain, and studies that investigated clinical populations without reporting comparison data with a typically developing control group. We excluded studies whose primary focus was to investigate a comorbid disorder (e.g., individuals with ADHD and Autism Spectrum Disorder) and studies focused on quantitative dimensions of reading or ADHD symptomatology without clearly identified dyslexic or ADHD groups. We excluded studies of prereaders at risk for dyslexia because our interest was in cases with confirmed dyslexia, and we excluded one study of preschoolers with ADHD because it was the only study in this early age range. We excluded two consortium studies, one for ADHD [55] and one for dyslexia [47], because they likely included participant overlap with existing studies (see Fig. 1 for a flow chart of screening procedures).

Nine studies that otherwise met criteria were not included in the meta-analysis because they did not report any group differences (two for dyslexia, [47, 56]; seven for ADHD, [57–63]). These null results do not contribute to the overall meta-analysis because the anatomic likelihood estimate (ALE) method tests for spatial convergence of foci across studies against the null hypothesis of random spatial convergence. As such, null results do not impact our coordinate-based meta-analysis in the same way as a traditional behavioral meta-analysis.

Table 1 lists the 37 studies that met inclusion criteria, with 22 investigating ADHD and 15 investigating dyslexia (see Additional file 2: Table S2 for expanded description). In the ADHD studies, 24 separate group contrasts were entered into the meta-analysis, and 18 different group contrasts were included for the studies investigating dyslexia. These numbers are consistent with guidelines for the number of studies needed for sufficient power (*N* = 17–20) in neuroimaging meta-analyses [101]. We opted not to restrict studies further by sample size requirements or study-specific statistical correction thresholds in order to be maximally inclusive of the existing VBM literature.

**Table 1:** Characteristics of studies included in the meta-analysis

Study (reference)	Total *N*	Age group analysis	Clinical group			Control group			Included in brain volume corrected analysis	Comorbidity with dyslexia or ADHD noted in exclusion criteria
			*N*	% male	Mean age (years)	*N*	% male	Mean age (years)		
ADHD										
Ahrendts et al. [64]	62	Adult	31	65	31.2	31	65	31.5	Yes	No learning disability
Bonath et al. [65]	36	-	18	100	13.6	18	100	14.1	Yes	-
Bralten et al. [66]	503	-	307	68	17.1	196	51	16.7	No	No learning disability
Brieber et al. [67]	30	-	15	100	13.1	15	100	13.3	Yes	-
Carmona et al. [68]	50	Child	25	84	10.8	25	84	11.2	Yes	-
He et al. [69]	72	Child	37	100	9.9	35	100	10.7	Yes	-
Iannaccone et al. [70]	40	-	20	61	14.5	20	50	14.8	Yes	-
Johnston et al. [71]	68	-	34	100	12.5	34	100	13.2	No	-
Kappel et al. (adults) [72]	36	Adult	16	94	23.5	20	100	23.7	No	-
Kappel et al. (children) [72]	24	Child	14	71	9.8	10	80	11.0	No	-
Kaya et al. [73]	37	Child	19	71	10.3	18	67	10.2	No	-
Kobel et al. [74]	26	Child	14	100	10.4	12	100	10.9	Yes	-
Kumar et al. [75]	36	Child	18	100	9.6	18	100	9.7	Yes	No learning disability
Lim et al. [76]	58	-	29	100	13.8	29	100	14.4	No	No dyslexia
McAlonan et al. [77]	59	Child	28	100	9.9	31	100	9.6	Yes	-
Montes et al. [78]	40	Adult	20	50	29.0	20	50	27.6	No	-
Moreno-Alcazar et al. [79]	88	Adult	44	66	31.6	44	66	32.6	No	-
Overmeyer et al. [80]	34	Child	18	83	10.4	16	94	10.3	Yes	No learning disability
Roman-Urrestarazu et al. [81]	83	Adult	49	76	22.2	34	50	22.9	No	-
Sasayama et al. [82]	35	Child	18	72	10.6	17	71	10.0	Yes	No learning disability
Van Wingen et al. [83]	29	Adult	14	100	32.0	15	100	37.0	Yes	-
Villemonteix et al. (med naïve group) [84]	57	Child	33	55	10.3	24	50	10.0	No	-
Villemonteix et al. (med group) [84]	44	Child	20	80	10.4	24	50	10.0	No	-
Yang et al. [85]	114	Child	57	61	11.1	57	60	11.7	Yes	-
Totals or sample size-weighted averages	1661		898	76	16.5	763	71	16.6		
Dyslexia										
Brambati et al. [86]	21	-	10	50	31.6	11	45	27.4	Yes	No psychiatric
Brown et al. [87]	30	Adult	16	100	24.0	14	100	matched to clinical grp	No	No ADHD
Eckert et al. [88]	26	Child	13	100	11.4	13	100	11.3	Yes	No psychiatric
Evans et al. (male adults) [89]	28	Adult	14	100	42.9	14	100	41.1	Yes	No severe psychiatric
Evans et al. (female adults) [89]	26	Adult	13	0	34.0	13	0	27.9	Yes	No severe psychiatric
Evans et al. (male children) [89]	30	Child	15	100	9.6	15	100	8.3	Yes	No severe psychiatric
Evans et al. (female children) [89]	34	Child	17	0	10.1	17	0	9.1	Yes	No severe psychiatric
Hoeft et al. [90]	38	-	19	53	14.4	19	53	14.4	Yes	No psychiatric
Jednoróg et al. [91]	236	Child	130	57	10.3	106	48	10.2	Yes	No ADHD
Kronbichler et al. [92]	28	-	13	100	15.9	15	100	15.5	Yes	No psychiatric
Liu et al. [93]	36	Child	18	72	11.8	18	83	11.8	Yes	No ADHD
Silani et al. [94]	64	Adult	32	100	24.4	32	100	26.3	No	-
Siok et al. [95]	32	Child	16	50	11.0	16	81	11.0	Yes	No ADHD
Steinbrink et al. [96]	16	Adult	8	75	20.1	8	75	23.7	Yes	No psychiatric
Tamboer et al. [97]	94	Adult	37	16	20.6	57	12	20.3	Yes	No ADHD
Vinckenbosch et al. [98]	23	Adult	13	100	Adults	10	100	Adults	Yes	No ADHD
Xia et al. [99]	48	Child	24	58	12.5	24	50	12.5	No	No psychiatric
Yang et al. [100]	23	Child	9	33	12.6	14	43	12.3	Yes	No ADHD
Totals or Sample size-weighted averages	833		417	61	16.4	416	57	16.5		

### Sample overlap

To examine sample overlap, we identified author overlap in papers for dyslexia or ADHD. For papers where there were overlapping authors, we examined the methods section for indications of sample overlap and for distinguishing features such as age range, recruitment source, or image acquisition parameters. The methods section of Jednoróg et al. [91] indicated partial overlap with a previous paper by Jednoróg et al. [102] which was removed from the analysis. In cases of ambiguity, we reached out to authors for clarification. Based on this correspondence, we removed Krafnick et al. [103] because of partial overlap with Evans et al. [104]. At the time of submission, there was an unresolved question of partial overlap between Brieber et al. [67] (*N* = 15 children with ADHD) and Johnston et al. [71] (*N* = 34 children with ADHD). There was no indication of sample overlap in the methods and a large time span between publications, so we included both studies in the final meta-analysis. However, out of an excess of caution, we re-ran the main conjunction analysis dropping the Brieber et al. study and confirmed the primary result was stable, only showing trivial changes in cluster size and ALE values (right caudate conjunction, *k* = 104 vs. 112, ALE 8.36 × 10^−3^ vs. 8.48 × 10^−3^, MNI coordinates *x* = 10, *y* = 14, and *z* = 8).

### Comorbid disorders in included studies

In ADHD, the most commonly reported comorbid disorders were anxiety disorders, oppositional defiant disorder, conduct disorder, and obsessive-compulsive disorder (Additional file 2: Table S2). Most ADHD studies (16 of 22, 73%) did not comment on dyslexia or learning disabilities in their exclusion criteria. Only three studies explicitly reported comorbidities with learning disabilities/dyslexia in their participants: 1 child with dyslexia of 18 ADHD cases [80], 1 child with dyslexia of 34 cases [71], and 5 children with learning disabilities of 57 ADHD cases [85].

The majority of dyslexia studies excluded all psychiatric disorders, with 7 of 15 (47%) specifically noting that participants with ADHD were excluded (Table 1). It is not clear if all authors considered ADHD in their screening of psychiatric disorders, especially since some studies noted only “severe psychiatric disorders.” None of the studies reported cases with comorbid ADHD in their samples.

These patterns indicate that the neuroimaging literature has generally taken a “pure cases” approach to recruitment. Based on our assessment of the existing studies, we find it more likely that the ADHD sample has undetected dyslexia comorbidity than vice versa*,* based on the screening procedures (see Table 1; Additional file 2: Table S2).

### Anatomic likelihood estimate (ALE) meta-analysis

The ALE meta-analysis method for neuroimaging studies, originally described by Turkeltaub et al. [105], uses a coordinate-based meta-analytic strategy. It treats each set of reported peak coordinates as the center of a probability distribution, in order to deal with inter-study differences in scanning parameters and imaging analyses. Newer versions of GingerALE software (version 2.3.6, www.brainmap.org/ale, [106–108]) incorporate random effects analysis to look for convergence between experiments. This procedure also adjusts the size of the Gaussian filter for the foci based on the number of participants in a study; smaller studies are blurred with a larger full-width half-maximum (FWHM) size than larger studies (e.g., foci emerging from a study with 10 participants have a 10-mm FWHM applied, as compared with a study of 50 participants, in which a 8.75-mm FWHM is applied). We used the analysis option that limits the effects of any single experiment on the ALE results [108].

Text files were generated that contained the gray matter (GM) foci reported in each study for the clinical group vs. typically developing (TD) group comparison, with separate files for each clinical group>TD and clinical group<TD. This yielded four separate analyses: ADHD>TD, ADHD<TD, dyslexia>TD, and dyslexia<TD. Coordinate foci files entered into this meta-analysis are published with this article (see Additional files 4, 5, 6 and 7). A conjunction analysis was used to evaluate the regions where the clinical groups show similar structural differences compared to the TD groups. Foci in Talairach space were converted to MNI space using the appropriate transform depending on the original data analysis: the relevant tal2icbm transform [109] was applied to foci that were analyzed in SPM or FSL, and foci that were reported in Talairach space that had been transformed from MNI space using the Brett transform were converted back to MNI space using the Brett transform (tal2mni). When foci were located outside the mask for the analysis, coordinates were adjusted to both fit within the mask and to conform to the anatomical region identified in the original publication. In this case, only one set of coordinates from Hoeft et al. [90] required adjustment of the *x* coordinate from 73.7 to 70 (shift of 3.7 mm), which is in the mean range of the adjustment performed in Fox et al. [110]. As noted above, the foci were blurred with a full-width half-maximum (FWHM) calculated based on the sample size of each study. A modeled activation (MA) map was created using the foci of each study by taking the maximum across each focus’ Gaussian [108], and the ALE image represents the union of all the MA maps. The null distribution of the ALE statistic at each voxel was then determined [107].

### Analyses

#### Within-disorder ALE analyses

First, the ALE maps representing coordinates from the ADHD vs. TD and dyslexia vs. TD studies were generated at two thresholds (1) an a priori more conservative threshold: *p* < .001 (uncorrected) with a minimum cluster size (*k*) of 50, and (2) a post-hoc more lenient threshold: *p* < .005 (uncorrected), *k* = 50. This yielded four ALE maps (dyslexia<TD, dyslexia>TD, ADHD<TD, and ADHD>TD) that highlight the regions where the literature indicates GM differences in each disorder (Tables 2 and 3). Because these ALE maps were being used as input to a conjunction analysis with its own statistical correction parameters (described next), we used uncorrected ALE maps at this step to ensure that we did not miss any potential areas of convergent GM differences by thresholding the ALE maps too strictly at this first stage. We selected *p*_uncorrected_ < .001 (*k* = 50) as an a priori threshold and then relaxed the threshold post-hoc to *p*_uncorrected_ < .005 (*k* = 50) to ensure that we did not miss any potential areas of conjunction that could be hypothesis-generating for future work, given that this is the first meta-analysis of dyslexia/ADHD gray matter overlap. We note throughout the manuscript which findings met our more conservative and more lenient thresholds for statistical significance.

**Table 2:** Gray matter differences in ADHD (*p* < .001, *k* = 50)

	Cluster #	Volume (mm^3^)	ALE Value	*x*	*y*	*z*	Label
ADHD<TD	1	552	0.013433	26	6	6	Right putamen
	2	272	0.014215	− 58	6	− 2	Left superior temporal gyrus
	3	144	0.011178	16	− 32	44	Right cingulate gyrus
	4	120	0.011226	− 8	− 10	48	Left cingulate gyrus
	5	88	0.010493	− 22	− 4	− 26	Left amygdala
	6	56	0.01083	− 26	16	− 24	Left inferior frontal gyrus
	7	56	0.010126	10	30	− 20	Right medial frontal gyrus / gyrus rectus
	8	56	0.010821	2	22	− 2	Right caudate head
	9	56	0.010804	28	70	− 2	Right superior orbitofrontal gyrus
	10	56	0.010802	− 14	52	14	Left superior frontal gyrus
	11	56	0.010811	− 40	− 6	56	Left precentral gyrus
ADHD>TD	1	160	0.007913	33	− 76	4	Right mid-occipital gyrus
	2	160	0.007913	− 14	− 84	37	Left cuneus
	3	160	0.007913	21	− 42	54	Right precuneus
	4	160	0.007913	− 17	12	58	Left superior frontal gyrus
	5	160	0.007918	− 6	− 20	66	Left paracentral lobule
	6	152	0.008282	− 26	− 28	70	Left postcentral gyrus
	7	152	0.00817	6	− 10	64	Right supplementary motor area
	8	144	0.007837	− 14	− 38	60	Left precuneus
	9	96	0.007112	− 2	− 15	5	Left thalamus, medial dorsal nucleus
	10	96	0.007271	− 13	− 27	40	Left cingulate gyrus
	11	96	0.007665	30	− 7	69	Right precentral/superior frontal gyrus
	12	96	0.007665	27	− 35	74	Right postcentral gyrus
	13	72	0.006635	− 16	− 34	68	Left postcentral gyrus
	14	64	0.007051	− 49	− 21	23	Left insula
	15	64	0.007051	− 15	− 45	37	Left posterior cingulate/precuneus
	16	64	0.006901	45	− 15	37	Right postcentral gyrus
	17	56	0.007732	− 14	− 54	46	Left precuneus
	18	56	0.007732	− 34	− 34	48	Left postcentral gyrus

**Table 3:** Gray matter differences in dyslexia (*p* < .001,* k* = 50)

	Cluster #	Volume (mm^3^)	ALE value	*x*	*y*	*z*	Label
Dyslexia<TD	1	336	0.010974	− 48	− 46	28	Left supramarginal gyrus
	2	312	0.011585	− 56	8	− 16	Left superior temporal gyrus
	3	192	0.010177	− 26	− 50	− 32	Left cerebellum lobule VI
	4	104	0.008865	36	− 64	− 10	Right inferior occipital gyrus
	5	96	0.009758	− 5	− 20	5	Left thalamus, medial dorsal nucleus
	6	96	0.008935	− 14	14	6	Left caudate body
	7	80	0.009177	38	47	− 12	Right orbitofrontal gyrus
	8	80	0.008973	− 56	− 52	2	Left middle temporal gyrus
	9	80	0.009207	52	− 52	22	Right superior temporal / supramarginal gyrus
	10	80	0.009177	20	39	40	Right superior frontal gyrus
	11	64	0.008617	− 48	− 26	22	Left insula
	12	56	0.009506	10	14	8	Right caudate body
Dyslexia>TD	1	576	0.009831	14	− 48	44	Right precuneus
	2	280	0.008859	− 57	− 53	42	Left inferior parietal lobule
	3	224	0.009177	− 32	− 76	− 23	Left cerebellum crus I
	4	224	0.009177	50	− 7	− 12	Right superior temporal gyrus
	5	224	0.008969	− 60	− 60	5	Left middle temporal gyrus
	6	152	0.007361	− 6	50	18	Left medial superior frontal gyrus
	7	152	0.007361	6	12	54	Right supplementary motor area
	8	96	0.006936	12	51	5	Right medial superior frontal gyrus
	9	96	0.006936	20	11	51	Right medial frontal gyrus
	10	80	0.007146	− 50	− 26	3	Left superior temporal gyrus
	11	80	0.007146	56	0	23	Right precentral gyrus
	12	80	0.007146	16	− 38	60	Right paracentral lobule
	13	80	0.006447	16	− 18	66	Right precentral gyrus

#### Conjunction analysis

Second, to determine any statistically significant overlap between areas of reduced GM in both ADHD and dyslexia, we conducted a conjunction analysis for the ADHD<TD and dyslexia<TD results using the more conservatively (*p*_uncorrected_ < .001, *k* =50) and more leniently (*p*_uncorrected_ < .005, *k* =50) thresholded maps. The conjunction analysis was thresholded at a false discovery rate (FDR) of *p* < .05 (estimated with 5000 permutations of the pooled dataset) with a minimum cluster size of 50. We did not conduct a conjunction analysis for the ADHD>TD and dyslexia>TD output, because visual inspection of both thresholded maps showed no evidence of overlap between the ADHD>TD and dyslexia>TD maps.

#### Impact of total brain volume

To evaluate the robustness of the main conjunction results, we ran a follow-up analysis that only included studies which (1) covaried for total brain volume or total gray matter volume or (2) explicitly tested for differences in total brain volume or total gray matter volume between groups and found null results. This follow-up analysis ensured that individual ALE maps for dyslexia and ADHD represented the most robust regionally specific findings in these literatures. Of the ADHD studies, 13 of 22 accounted for total brain or gray matter volume. Of the dyslexia studies, 12 of 15 accounted for total brain or gray matter volume (see Table 1).

#### Impact of age

We examined the potential impact of age on case-control GM differences by repeating the analyses with studies grouped based on whether the participants were children (mean of clinical and control group ages between 6 years, 0 months, and 12 years, 11 months; no adults included in the study) or adults (18 years and up). Studies that included both children and adults in the sample were not included in this sub-analysis. To our knowledge, the GingerALE software does not include functionality to test moderation directly, so we proceeded by analyzing these age-based subgroups separately. Among the ADHD group contrasts, 12 met our inclusion criteria for the child analysis while 6 investigated adult participants. Among the dyslexia group contrasts, 8 studies met our inclusion for the child analysis, while 7 investigated adult participants (see Table 1 for designation of which studies were included in the child or adult analysis). There were not enough studies to create a separate adolescent age group.

The ALE maps for ADHD<TD_children_, ADHD<TD_adults_, dyslexia<TD_children_, and dyslexia<TD_adults_ were generated and thresholded at the same more conservative (*p*_uncorrected_ < .001, *k* = 50) and more lenient (*p*_uncorrected_ < .005, *k* = 50) thresholds. For the age analysis, the number of studies reporting increased GM in both disorders were too few to conduct meaningful analyses. As in the main analysis, a conjunction analysis of the child dyslexia and ADHD maps and the adult dyslexia and ADHD maps was conducted using FDR *p* < .05 (estimated with 5000 permutations of the pooled dataset) with a minimum cluster size of 50.

#### Data visualization and reporting

The data were visualized using MRIcroGL (http://www.cabiatl.com/mricrogl/) with the thresholded ALE maps as the overlay and the MNI152 brain as the underlay. The size, extent, peak coordinates, and ALE values for each statistically significant cluster are reported in Tables 2 and 3.

## Results

### Gray matter differences in ADHD

**Fig. 2:**
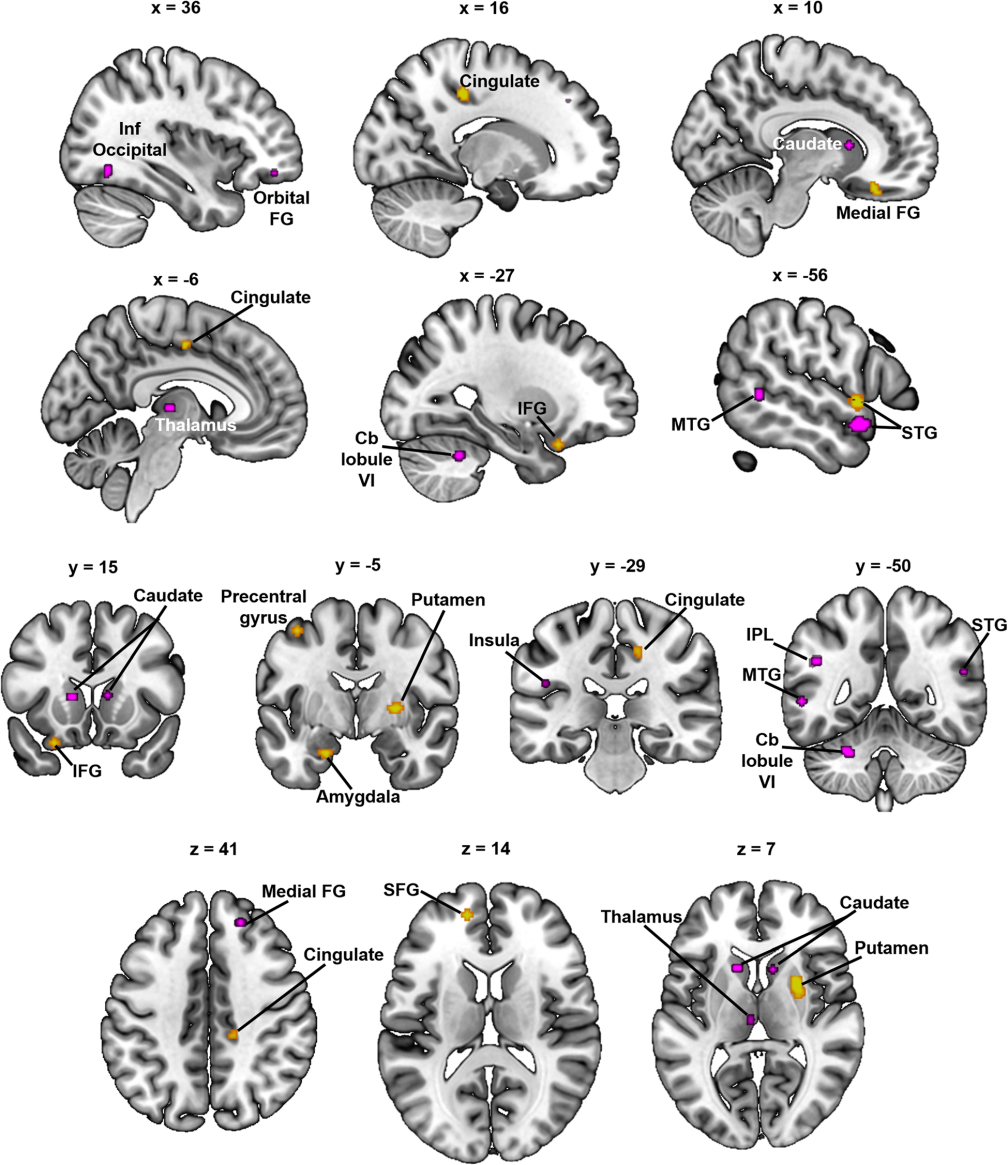
Decreased gray matter in ADHD and dyslexia. Regions of statistically significant ALE values (*p*_uncorrected_ < .001, *k* = 50) indicating decreased GM in ADHD vs. TD (yellow-orange) and dyslexia vs. TD (violet) are shown on the same template. FG frontal gyrus, Cb cerebellum, IFG inferior frontal gyrus, Inf inferior, MTG middle temporal gyrus, STG superior temporal gyrus, IPL inferior parietal lobule, SFG superior frontal gyrus

**Fig. 3:**
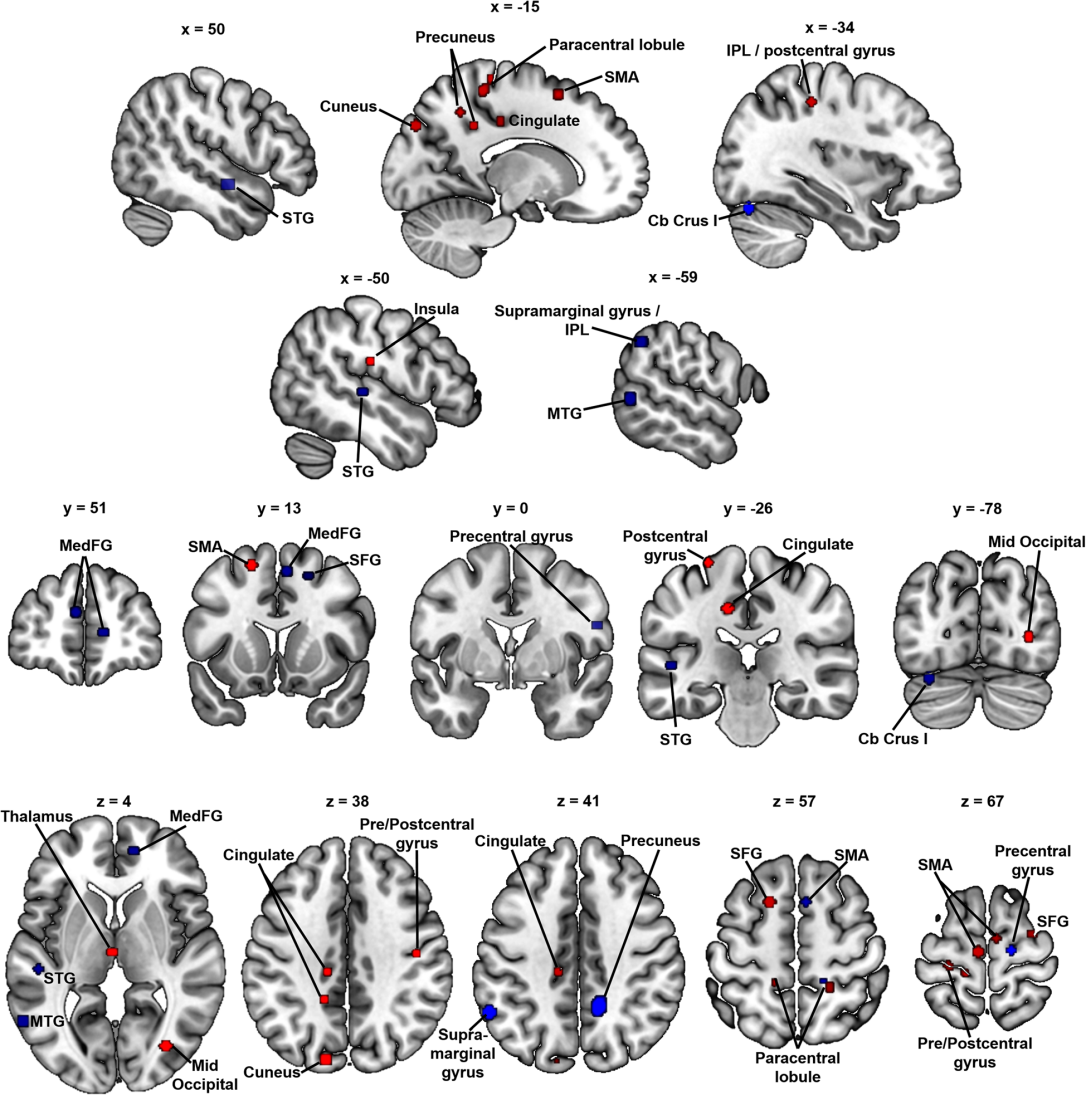
Increased gray matter in ADHD and dyslexia. Regions of statistically significant ALE values (*p*_uncorrected_ < .001, *k* = 50) indicating increased GM in ADHD vs. TD (red) and dyslexia vs. TD (blue) are shown on the same template. MTG middle temporal gyrus, SMA supplementary motor area, IPL inferior parietal lobule, Cb cerebellum, STG superior temporal gyrus, med medial, mid middle, FG frontal gyrus, SFG superior frontal gyrus

Table 2 provides the details of regions in which participants with ADHD showed differences in GM relative to a typically developing comparison group at the more conservative *p* < .001, *k* =50 threshold. Reduced GM in ADHD was evident in the right basal ganglia (caudate and putamen), left superior temporal gyrus, cingulate cortex, left amygdala, and several frontal cortical regions (Fig. 2, yellow-orange). Increased GM in ADHD was found in areas associated with sensorimotor planning and execution (supplementary motor area, pre- and postcentral gyri), the thalamus, as well as occipital (middle occipital gyrus) and parietal (posterior cingulate, cuneus, precuneus) areas (Fig. 3, red).

### Gray matter differences in dyslexia

Table 3 shows regions where the ALE analyses showed GM differences in dyslexia at the more conservative *p* < .001, *k* =50 threshold. Reduced GM was evident in dyslexia in left-hemisphere middle and superior temporal regions, inferior parietal regions, and cerebellum (lobule VI); right medial and orbital frontal regions; and the caudate bilaterally (Fig. 2, violet). Increased GM in dyslexia compared with controls was evident in the left supramarginal gyrus/inferior parietal lobule, middle temporal gyrus, and cerebellum (Crus I); right precuneus, supplementary motor area, and precentral gyrus; and medial frontal regions (Fig. 3, blue).

### Conjunction analysis

**Fig. 4:**
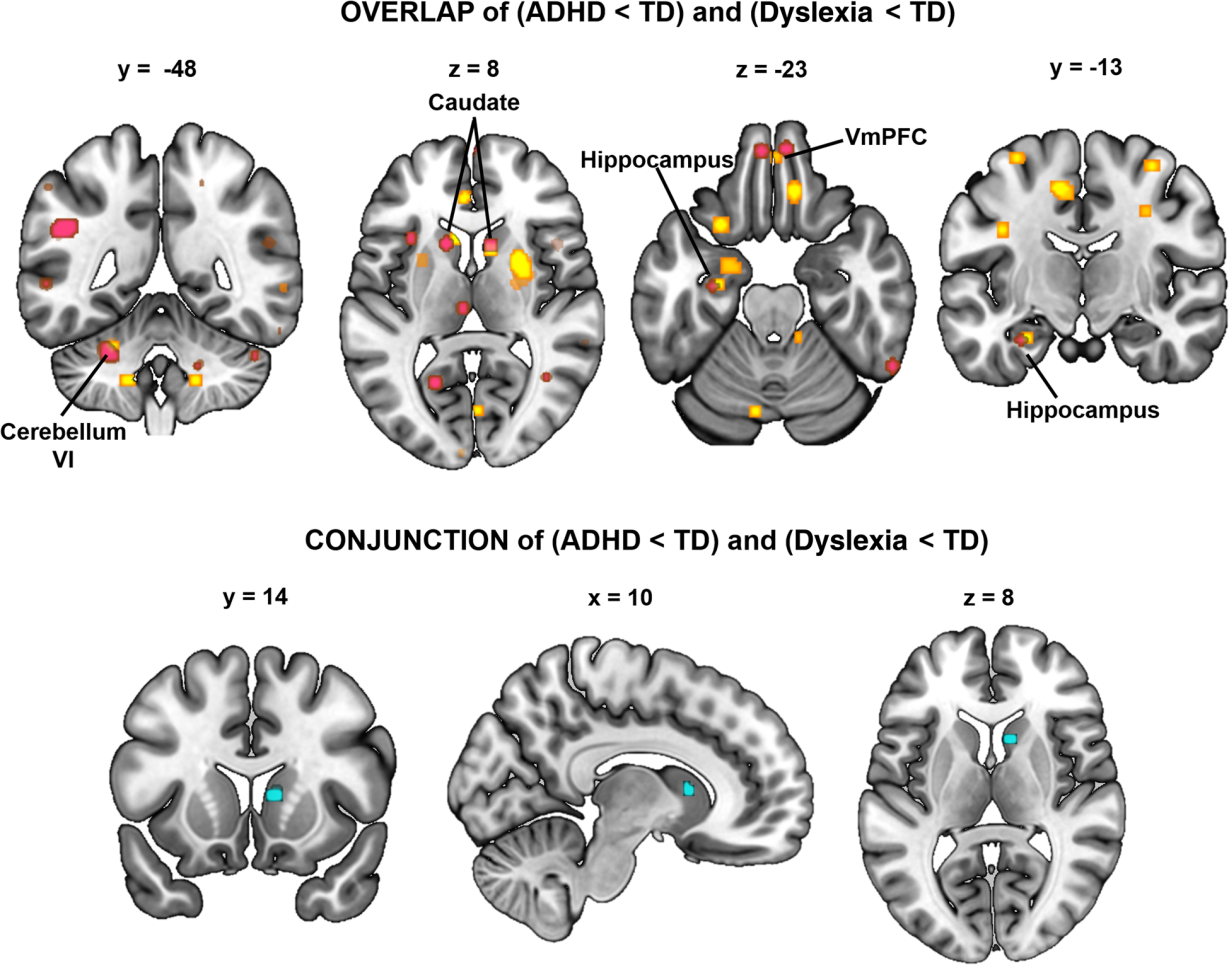
Conjunction of the ADHD<TD and developmental dyslexia<TD ALE maps. The top two rows show the ADHD<TD (yellow-orange) and dyslexia<TD (violet) ALE maps at the lenient threshold of *p*_uncorrected_ < .005, *k* = 50. There is visual overlap between the maps in the caudate bilaterally, left hippocampus, left cerebellum, and bilateral vmPFC. Results of the statistical conjunction analysis (FDR *p* < .05, *k* = 50) reveal overlap in the right caudate (cyan, bottom row). vmPFC ventromedial prefrontal cortex

We tested for regions of overlap between the areas of GM reduction in ADHD and dyslexia using both the more conservatively (*p*_uncorrected_ < .001, *k* = 50) and more leniently thresholded (*p*_uncorrected_ < .005, *k* = 50) ALE maps. There was no statistically significant conjunction of the ALE maps using the more conservative threshold (*p* < .001, *k* = 50). Figure 4 shows the ALE maps at the more lenient threshold (*p* < .005, *k* =50). While there was some visual overlap in the caudate bilaterally, left hippocampus, left cerebellum, and bilateral ventromedial prefrontal cortex (vmPFC), the statistical conjunction analysis (FDR *p* < .05, *k* = 50, 5000 permutations) revealed that only the right caudate survived statistical correction (*k* = 112, ALE 8.48 × 10^−3^, MNI coordinates *x* = 10, *y* = 14, and *z* = 8; see Fig. 4).

GingerALE does not have a quantitative way to determine how individual studies contribute to a conjunction result, so we used visual inspection of the ALE maps and evaluation of the reported coordinates to investigate the conjunction result. While there were several studies of dyslexia and ADHD that reported coordinates in the right caudate, the studies that reported coordinates closest to the conjunction peak were the Yang et al. [85] ADHD study (*x* = 10, *y* = 12, *z* = 7) and the Tamboer et al. [97] dyslexia study (*x* = 10, *y* = 14, z = 8). Notably, the Tamboer et al. dyslexia study specifically excluded comorbid ADHD, so it is unlikely that high rates of comorbid ADHD in the dyslexia sample can explain the conjunction. Both the Yang (*n* = 114) and Tamboer (*n* = 94) studies were the second largest VBM studies in their respective literatures.

### Impact of total brain volume

To test the robustness of the conjunction in the right caudate, we re-ran the conjunction analyses excluding studies that did not correct for total brain volume (see Table 1). This analysis used the more leniently thresholded ALE maps (*p*_uncorrected_ < .005, *k* = 50). The right caudate remained the only statistically significant region of conjunction between ADHD<TD and dyslexia<TD maps (FDR *p* < .05, 5000 permutations; *k* = 120, ALE 8.48 × 10^−3^, MNI coordinates *x* = 10, *y* = 14, *z* = 8).

### Impact of age

When analyses were restricted to studies of dyslexia and ADHD in adults, there was no overlap in reduced GM at either the conservative (*p*_uncorrected_ < .001, *k* = 50) or liberal (*p*_uncorrected_ < .005, *k* = 50) thresholds. In children, there was no overlap between the regions showing less GM in the clinical groups relative to the TD groups at *p*_uncorrected_ < .001. When the maps were thresholded at *p*_uncorrected_ < .005, there was a small cluster in the left middle frontal gyrus/supplementary motor area where there was overlap between reduced GM in both groups (*k* = 64, ALE 6.75 × 10^−3^, MNI coordinates *x* = − 28, *y* = 19, *z* = 43) (see Additional file 3: Table S3).

## Discussion

This study presents the first meta-analysis of overlap in gray matter differences between dyslexia and ADHD. The rationale for this “conjunction” approach to the meta-analysis is derived from existing multiple deficit models of dyslexia and ADHD [3, 7, 13, 14]. In these conceptualizations, the comorbidity of dyslexia and ADHD is believed to arise, at least partly, from shared genetic factors that may manifest in shared cognitive risks, such as processing speed [13, 18] and executive functions [17]. The current study fills a gap at the neural level of analysis by attempting to identify overlapping gray matter correlates associated with both disorders.

A general theme emerging from the results of this meta-analysis is that there is a surprising lack of overlap between the disorders. The same pattern was true when we restricted the analyses to age-specific comparisons for children and adults. While there were isolated findings that emerged using our lenient thresholds, it was notable that the overall pattern was one of the distinctiveness of gray matter correlates in dyslexia and ADHD. Here, we discuss (1) the state of the VBM literature in both disorders, (2) regions of convergence, and (3) why shared neural correlates may have been difficult to find.

### VBM literature in dyslexia and ADHD

A precondition for examining overlapping structural differences in dyslexia and ADHD is that the individual literatures are sufficiently advanced to show good convergence within disorder before cross-disorder convergence can be assessed. Because both literatures have had replication difficulties [39, 42], we will first consider the correspondence of our disorder-specific results with previous meta-analyses.

#### Meta-analyses of VBM studies in dyslexia

There have been three meta-analyses of VBM studies in dyslexia [47–49]. Richlan et al. [49] and Linkersdorfer et al. [48] each included 9 studies, while Eckert et al. [47] included 11 studies. The overlap in the studies included in previous meta-analyses and the current meta-analysis ranges from 46%–53%. Richlan et al. [49] reported gray matter reduction in the right superior temporal gyrus and the left superior temporal sulcus; regional gray matter reductions in left ventral occipitotemporal and left cerebellar regions were too scattered for reliable meta-analytic clustering. Linkersdorfer et al. [48] reported gray matter reductions in the left fusiform gyrus (extending into the left inferior temporal gyrus), bilateral supramarginal gyrus (right cluster extended to the posterior portion of the superior temporal gyrus), and bilateral cerebellum (lobule VI). The most recent meta-analysis by Eckert et al. [47] reported gray matter reductions in left orbitofrontal cortex/inferior frontal gyrus, left posterior superior temporal sulcus/middle temporal gyrus, and right cerebellum. The Eckert et al. study also included a direct VBM analysis of the largest consortium dataset to date (*N* = 164 children with dyslexia; *N* = 129 controls). In contrast to the meta-analytic results, the direct analysis did not detect any statistically significant regions of reduced gray matter after controlling for total gray matter volume [47]. Taken together, across studies there is some consistency in areas implicated by at least two of the meta-analyses, including left superior temporal/temporoparietal regions, left ventral occipitotemporal regions, right superior temporal regions, and bilateral cerebellar regions. These findings in the left hemisphere show good convergence with the two posterior neural systems in the left hemisphere that have been repeatedly implicated in dyslexia. Our meta-analytic results were consistent with previous meta-analyses in the left and right temporoparietal regions and left cerebellar lobule VI. However, we also note the null findings from Eckert et al.’s [47] consortium VBM analysis which suggests that this literature continues to have inconsistencies [39] that should be addressed by larger imaging samples and continued meta-analytic strategies.

#### Meta-analyses of VBM studies in ADHD

The ADHD VBM literature has been meta-analyzed in four previous studies [32, 40, 50, 51]. The overlap in the studies included in previous meta-analyses and the current meta-analysis ranges from 18% study overlap with the earliest meta-analysis [50] to 68% study overlap with the most recent meta-analysis [﻿32﻿]. The first meta-analysis included 7 studies [50] and found gray matter reductions in the right putamen/globus pallidus in individuals with ADHD compared to controls. Nakao et al. [51] included 14 studies, and the most robust finding was reduced gray matter volume in the right basal ganglia, including the putamen, globus pallidus, and the caudate nucleus. Frodl et al. [40] included 11 studies, and also reported reduced gray matter in the basal ganglia (right globus pallidus, right putamen) as well as bilaterally in the anterior cingulate cortex (ACC). The most recent meta-analysis by Norman et al. [﻿32﻿] (27 studies) showed decreased gray matter in the right putamen/pallidum/insula, right caudate nucleus, ventromedial orbitofrontal cortex/ventromedial prefrontal cortex/rostral ACC, and left occipital lobe. Taken together, regions in right basal ganglia structures and ACC are consistently reduced in ADHD across studies, which is in line with hypotheses of fronto-striatal dysfunction in ADHD. Likewise, our results showed reduced gray matter in right basal ganglia structures (putamen, caudate) and medial frontal regions.

### Potential regions of overlap

In the primary conjunction analysis, there was no statistically significant conjunction between our more conservatively thresholded ALE maps (*p* < .001, *k* = 50), but decreased gray matter in the right caudate emerged as a region of statistically significant conjunction between dyslexia and ADHD when using our leniently thresholded ALE maps (*p* < .005, *k* = 50). This overlap in the caudate remained significant in our follow-up analysis of studies accounting for total brain volume, suggesting that this regional difference is specific and not attributable to global structural differences. These results suggest that this region is worth further exploration regarding its potential relevance to ADHD and dyslexia. In fact, our confidence in this finding has increased due to a recently published paper reporting converging results [30]. Jagger-Rickels et al. [30] recently published the first VBM study of comorbid dyslexia+ADHD where they compared children with dyslexia only (*N* = 17), ADHD only (*N* = 41), and dyslexia+ADHD (*N* = 16) to controls (*N* = 32). They reported that regions of the right caudate showed smaller volumes in all three clinical groups, consistent with the results of this meta-analysis and the notion of the right caudate as a shared neural correlate of both disorders.

In ADHD, the caudate has been a long-standing region of interest in both structural and functional neuroimaging studies as a critical component of frontal-striatal circuits implicated in ADHD (i.e., [111]). Decreases in caudate volume in ADHD are one of the most consistent structural findings reported in ADHD [32, 40, 50–52]. The caudate also shows functional differences in ADHD. For example, a recent meta-analysis of fMRI studies of ADHD reported under-activation of the right caudate relative to controls during go/no-go tasks [41]. Structural and functional differences in the caudate could underpin executive function impairment in ADHD.

While striatal dysfunction has been a central focus of investigation in ADHD, it has only recently emerged as a region of interest in dyslexia [87, 97, 112]. Because of this, it is not clear how striatal structural differences might be related to dyslexia. Tamboer et al. [97] showed that the same region in the right caudate nucleus as we report in the current study (MNI *x* = 10, *y =* 14, *z* = 8) was significantly correlated (*r* = .61) with a rhyme/confusion factor. The rhyme/confusion factor includes a Dutch-English rhyming task [97]. The authors speculated that the correlation might be related to executive dysfunction, because the rhyming task required switching between languages. This interpretation is consistent with the notion that fronto-striatal dysfunction may be related to executive function deficits in both dyslexia and ADHD.

The striatum has also emerged as a region of interest in functional neuroimaging studies of dyslexia. Meta-analytic studies have reported consistent hyperactivation in several frontal-striatal regions, including the bilateral striatum (both putamen and caudate) [113–115]. These hyperactivations have been interpreted as “compensatory,” though specific mechanisms remain unclear. Hancock et al. [112] explored three specific hypotheses about these hyperactivations, given the role of the striatum in (1) articulatory processing, (2) phonological processing, and (3) implicit/procedural learning. They found the strongest level of support for overlap of dyslexia hyperactivations in fronto-striatal circuits with articulation functional maps, suggesting compensatory activity potentially related to subvocalizations during reading. While articulatory processes were the leading hypothesis based on their results, it is difficult to conclusively rule out the other hypotheses. The authors did not explore potential overlap with executive functioning maps, which also remains a competing hypothesis.

Taken together, the role of the caudate in dyslexia remains unclear, but executive functions and procedural learning are two candidate cognitive constructs that may overlap between dyslexia and ADHD. There is extensive neuropsychological evidence documenting executive dysfunction in both dyslexia and ADHD, especially in working memory, inhibition, and sustained attention, which depend on frontal-striatal circuitry. Procedural learning is a newer hypothesis that deserves further scrutiny [116, 117]. There is emerging evidence for procedural learning deficits in dyslexia, most notably from a meta-analysis of the most widely-used procedural sequence learning task, the serial reaction time task [118]. Procedural learning deficits have also been hypothesized in ADHD, partly because procedural learning depends on frontal-striatal circuitry. The evidence-base is small at present, but there are promising leads [119, 120]. In summary, the overlap between dyslexia and ADHD in the right caudate might point to impairments in procedural learning and/or executive functions that are risk factors for both disorders.

Our analyses of age-based subgroups showed an overlap in the left middle frontal gyrus/supplementary motor area between children with ADHD and children with dyslexia at our liberal statistical threshold. This analysis should be interpreted with caution because of the reduced number of contributing studies and the liberal statistical threshold. Nevertheless, we report this finding for hypothesis-generating purposes. A plausible interpretation of this region of overlap is again attributable to shared impairments in executive function in dyslexia and ADHD, due to the critical role of the frontal circuitry in executive functions [121–123], including working memory and inhibitory control.

### Evaluating potential explanations for minimal gray matter overlap

The foregoing discussion focused on regions of overlap, but the overall pattern of results was notable in the specificity of the gray matter correlates in dyslexia and ADHD. How can we understand the overall distinctiveness of the gray matter correlates of dyslexia and ADHD in the context of a strong genetic correlation between the two disorders, *r*_*g*_ ~ .50–.70 [7]? There are a few points to consider.

#### Comorbidity

First, it appears that the neuroimaging literatures of both disorders have generally sought to recruit “pure” groups. This recruitment strategy does not completely explain the lack of overlap, however, because we can infer from the genetic correlation that a genetic factor influencing dyslexia is also 50–70% likely to influence ADHD as well (and vice versa). Since both dyslexia and ADHD are known to be complex polygenic disorders likely involving hundreds to thousands of genes [124, 125], many children with “pure” dyslexia and “pure” ADHD should possess a number of genetic risk factors that could be considered “shared” and we would expect these shared genetic factors to influence shared neural systems as well. Thus, a comparison of “pure” disorders is actually the strongest test of the correlated liabilities model. In this context, the fact that we did identify a region of overlap in the right caudate, albeit at more lenient statistical thresholds, is an important hypothesis-generating finding for future work. While a “pure” disorders recruitment strategy may have attenuated the overlap of dyslexia and ADHD in our meta-analysis, we suggest that our main finding of distinctive gray matter differences in ADHD and dyslexia is not entirely attributable to recruitment approach.

#### Developmental considerations

Is it possible that our mainly null results could be due to mismatches in age recruitment between dyslexia and ADHD? While dyslexia studies included proportionally more adult samples than ADHD studies (*N* = 7 adult studies of 15 for dyslexia, *N* = 6 adult studies of 22 for ADHD), the sample size-weighted age comparisons indicate that age mismatches are unlikely to be a primary problem (dyslexia = 16.4 years; ADHD = 16.5 years). Moreover, our follow-up analysis restricting to just child samples and just adult samples where we continued to find largely distinctive patterns across disorders partially addressed this issue. However, with the increase in homogeneity of age, there is a corresponding decrease in sample size and power and so the null findings are less interpretable.

#### Alternative imaging modalities

It is possible that VBM is not sufficiently sensitive to detect the overlapping neural correlates of both disorders, which may be better indexed by methods designed to assess structural and functional connectivity or functional signatures under task demands. While there is evidence that gray matter alterations can be correlated with functional abnormalities, the overlap is not complete [48].

### Next steps

If gray matter alterations are not capturing the shared neurobiological risk associated with dyslexia and ADHD, what is the most promising direction for further studies of this question? One promising next step is to use the neuropsychological findings to inform neuroimaging studies of the overlap of these two disorders. For example, processing speed is a construct that has been associated with both disorders and can account for a substantial portion of the comorbidity or covariance (~ 75%) [13, 14]. Moreover, in a previous study, all of the shared genetic influences between reading and inattention symptoms were also shared with processing speed, indicating that processing speed may be a marker of the correlated genetic liability of the two disorders [7]. The most consistent neural correlate of processing speed is white matter volume and integrity, with broad involvement from frontal, parietal, and temporal regions [126]. These associations lead to the hypothesis that compromised white matter integrity may jointly increase risk for reading and attention problems via processing speed impairments. Further work on this hypothesis is needed through individual studies of potential overlapping white matter differences in these disorders.

In terms of the design of neuroimaging studies, there are important next steps to take in characterizing and recruiting comorbid samples to address both shared and specific features of dyslexia and ADHD. While most dyslexia samples screened out ADHD, most ADHD studies did not comment on comorbid dyslexia or learning disabilities. One first step is for neuroimaging studies of dyslexia and ADHD to directly assess ADHD and reading symptoms, respectively. Brief, standardized instruments are available to assess both domains. Direct assessments would be helpful because many studies in the existing literature used parent or self-report of co-occurring diagnoses, and so likely under-estimate the rate of true comorbidity. Direct assessments would also permit the investigation of subclinical variation in comorbid disorders, which is important given that both dyslexia and ADHD are conceptualized as extreme phenotypes on an underlying continuous distribution [127, 128].

The ideal recruitment strategy for investigating the neural correlates of the dyslexia-ADHD comorbidity is to collect individuals with dyslexia, ADHD, dyslexia+ADHD, and typically developing controls. Only a few studies have taken this approach (e.g., [30, 31]). In the past, such comorbid designs have been used to document differences, not similarities, between groups. However, the correlated liabilities model predicts that all three clinical groups should show similarities in some neural correlates, so it is important that analyses are designed to investigate shared as well as specific neural correlates.

### Limitations

The current results should be considered in light of a few limitations. As with any meta-analysis, our analysis is constrained by the design and statistical decisions of the primary studies. While the neuroimaging field is moving toward larger samples in general, Table 1 shows that it is still quite common to use sample sizes in the range of 20–30 individuals per group, which are likely underpowered for expected effect sizes [39, 42]. Given these power limitations, it remains possible that gray matter correlates with smaller effects have not been reliably detected, and some of these undetected correlates could be overlapping between dyslexia and ADHD.

Relatedly, the ALE meta-analytic approach relies on modeling the peak coordinates reported in studies and does not account for the extent of statistically significant findings (i.e., cluster size). It is possible that this approach leads to a more conservative estimation of potential sample overlap in the cases of studies reporting large clusters which extend well beyond the region that would be modeled by the ALE approach.

Recruitment across studies for dyslexia and ADHD was heterogeneous. For dyslexia, some studies included participants with a previous clinical diagnosis while others established their own empirical criteria on standardized reading measures. Similarly, for ADHD, studies varied in whether they employed clinical diagnoses, standardized diagnostic interviews and/or behavioral rating scales. These recruitment differences likely add to the heterogeneity of the clinical populations, potentially making it more difficult to identify consistent gray matter correlates within disorders, and thereby making it more difficult to discern overlaps between the disorders.

It was beyond the scope of this meta-analysis to examine medication effects in ADHD (for a review see [40]), but we note that medication may normalize structural differences in ADHD [51], though this is not a universal finding [42]. If medication does normalize structural differences, this might make it difficult to identify genetically driven overlaps between dyslexia and ADHD. Further studies could focus on the overlap of brain regions associated with family risk for dyslexia and ADHD in preschool children before the onset of reading and before stimulant initiation to more narrowly focus on neurobiological risk factors for both disorders, rather than the consequences of reduced reading experience and stimulant use.

Lastly, it is important to consider the role of publication bias in this meta-analysis. Analytic strategies for identifying publication bias in the neuroimaging literature are still emerging because of the unique challenges associated with this type of data (e.g., [129, 130]). For neuroimaging studies, there are related concerns for the role of “missing” null studies (i.e., the file drawer problem) and concerns for false positives in the published literature [131–133]. In our analysis, the problem of false positives is a larger threat to validity than the file drawer problem. In our coordinate-based meta-analytic framework, null studies do not influence the disorder-specific meta-analytic results because the method tests for spatial convergence of foci across studies against the null hypothesis of random spatial convergence. However, what would weaken the evidence for true convergence are studies that reported multiple false positives. Such random noise would diminish the statistical evidence for convergence of true effects across studies [129]. False positives are likely given the unique characteristics of the neuroimaging literature where there is high pressure to publish because of the expense of studies coupled with multiple decision points in the analysis and a high multiple testing burden [134]. In this case, we must consider the role of confirmation bias such that false-positive brain associations that are aligned with existing theories are more likely to be published. While we acknowledge the potential role of theory-aligned false positives in both the dyslexia and ADHD literature, we note that the conjunction analysis across the dyslexia and ADHD literatures is somewhat immune to this concern because these literatures have been quite theoretically distinct. It seems unlikely that false positives in both literatures would overlap to give a false positive conjunction. Of course, the most persuasive evidence will come from independent replication in well-powered samples, which shows some initial promise in the case of the right caudate finding [30].

## Conclusions

To our knowledge, the current study is the first to meta-analyze the overlap of gray matter correlates of dyslexia and ADHD. The overall pattern was one of largely distinctive gray matter correlates, although we identified a region of overlap in the right caudate when using our more lenient statistical thresholds. This overlap in the right caudate may be related to shared cognitive correlates in executive functions and/or procedural learning. Our goal was to identify shared gray matter differences in order to contribute to a multi-level understanding to the dyslexia-ADHD comorbidity that spans the genetic, neural, and cognitive levels of analysis. This framework is important not only for the dyslexia-ADHD comorbidity specifically, but also for the broader field of neurodevelopmental disorders where comorbidity is pervasive.

## Supplementary information

**Additional file 1.** Checklist for neuroimaging meta-analyses

**Additional file 2.** Detailed characteristics of studies included in the meta-analysis.

**Additional file 3.** Gray matter differences in ADHD and dyslexia in adults and children (p<.001, *k* =50)

**Additional file 4.** GingerALE meta-analysis script for ADHD > TD.

**Additional file 5.** GingerALE meta-analysis script for Dylexia > TD.

**Additional file 6.** GingerALE meta-analysis script for Dyslexia < TD.

**Additional file 7.** GingerALE meta-analysis script for ADHD < TD.

## Data Availability

Meta-analysis coordinates entered into the publicly available GingerALE software (http://www.brainmap.org/ale/) are provided as supplementary files (Additional files 4, 5, 6 and 7). These text files report the gray matter foci for existing dyslexia vs. controls and ADHD vs. controls voxel-based morphometry studies, with separate files for clinical group > controls and clinical group < controls

## Abbreviations

ACC: Anterior cingulate cortex
ADHD: Attention-deficit/hyperactivity disorder
ALE: Anatomic likelihood estimate
Cb: Cerebellum
FDR: False discovery rate
FG: Frontal gyrus
FWHM: Full-width half-maximum
GM: Gray matter
Inf: Inferior
IPL: Inferior parietal lobule
*k*: Cluster size
MA: Modeled activation
med: Medial
mid: Middle
MNI: Montreal Neurological Institute
MTG: Middle temporal gyrus
PRISMA: Preferred Reporting Items for Systematic Reviews and Meta-analyses
SFG: Superior frontal gyrus
SMA: Supplementary motor area
STG: Superior temporal gyrus
TD: Typically developing controls
VBM: Voxel-based morphometry
vmPFC: Ventromedial prefrontal cortex
