# Supplementary material for: Are there shared neural correlates between dyslexia and ADHD? A meta-analysis of voxel-based morphometry studies
Source: J Neurodev Disord. 2019 Nov 21;11:31. doi: 10.1186/s11689-019-9287-8 (PMC6873566; doi:10.1186/s11689-019-9287-8)
Supplement: Supplementary file 1 — Additional file 1. Checklist for neuroimaging meta-analyses [file 11689_2019_9287_MOESM1_ESM.docx]

Supplementary Table 1. Checklist for neuroimaging meta-analyses.

| The research question is specifically defined | Yes. Studies were included that reported results from direct group comparisons in gray matter between the clinical group (dyslexia, ADHD) and an age-matched typically-developing control group. The extracted coordinates were from the following contrasts:  Dyslexia > Controls  Dyslexia < Controls  ADHD > Controls  ADHD < Controls  This resulted in four ALE maps, two for Dyslexia (Dyslexia>Controls, Dyslexia<Controls) and two for ADHD (ADHD>Controls, ADHD<Controls).  We then conducted statistical conjunction analysis between the ALE maps for Dyslexia<Controls and ADHD<Controls to examine the overlap between the ALE maps for the two clinical groups. |
| --- | --- |
| The literature search was systematic | Yes, it included the following keywords in the following databases:  Pubmed:  *(Dyslexia [MeSH] OR dyslex* OR reading disab* OR reading disorder*) AND (“voxel-based” OR “voxel based” OR VBM OR “gray matter” OR “grey matter”) AND ("1999/01/01"[Date - Publication] : "2018/04/30"[Date - Publication]) AND English[Language]*  *(Attention Deficit Disorder with Hyperactivity [MeSH] OR ADHD OR attention*deficit) AND (“voxel-based” OR “voxel based” OR VBM OR “grey matter” OR “gray matter”) AND ("1999/01/01"[Date - Publication] : "2018/04/30"[Date - Publication]) AND English[Language]* |
| Detailed inclusion and exclusion criteria are included | No non-standard criterion |
| Sample overlap was taken into account | Yes, using the following method: Studies with overlapping authors were examined for independence of the samples by examining sample characteristics and image acquisition parameters and contacting study authors in ambiguous cases. |
| All experiments use the same search coverage (state how brain coverage is assessed and how small volume corrections and conjunctions are taken into account) | Yes, the search coverage is the following:  All included coordinates were extracted from studies conducting whole-brain analyses. Results from region of interest or conjunction analyses were not included in the meta-analysis. |
| Studies are converted to a common reference space | MNI: All coordinates that were reported in Talairach space were converted to MNI space for analysis. Different transforms were applied depending on the analysis procedures reported in each original study. GingerALE provides transformation options depending on the software package (e.g. SPM, FSL) or reported transform (e.g. Brett) used in the original study. Therefore, the tal2icbm transform was applied for studies reporting Talairach coordinates from SPM or FSL analyses, and studies reporting use of the Brett transform for conversion from MNI to Talairach space were converted back to MNI space using the Brett tal2mni transform. |
| Data extraction have been conducted by two investigators (ideal case) or double-checked by the same investigator (state how double-checking as performed) | Yes, the following authors:  CJS, LMM checked inclusion criteria  CJS, LMM, SP extracted coordinates  LMM, RS extracted phenotype info |
| The paper includes a table with at least the reference, basic study description (e.g., for fMRI tasks: stimuli), contrasts, basic sample descriptions (e.g., size, mean age, gender distribution, specific characteristics) of the included studies, source of information (e.g., contact with authors, reference space | Yes, and also the following data:  Comorbidities, whether total brain volume was controlled in analysis, inclusion in previous meta-analyses, table reporting peak coordinates |
| The study protocol was previously registered and all analyses planned beforehand, including the methods and parameters used for inference, correction for multiple testing etc. | The study protocol was not pre-registered, but post-hoc analyses are clearly stated in the paper. |
| The meta-analysis includes diagnostics | We examined the characteristics of the studies most strongly contributing to the conjunction result in the right caudate. |
