# Supplementary material for: Are there shared neural correlates between dyslexia and ADHD? A meta-analysis of voxel-based morphometry studies
Source: J Neurodev Disord. 2019 Nov 21;11:31. doi: 10.1186/s11689-019-9287-8 (PMC6873566; doi:10.1186/s11689-019-9287-8)
Supplement: Supplementary file 3 — Additional file 3. Gray matter differences in ADHD and dyslexia in adults and children (p<.001, k =50) [file 11689_2019_9287_MOESM3_ESM.docx]

**Supplementary Table 2. GM differences in ADHD and dyslexia in adults and children**

|  | Cluster # | Volume (mm^3^) | ALE Value | x | y | z | Label |
| --- | --- | --- | --- | --- | --- | --- | --- |
| **Adults: Dyslexia < TD** | Cluster # | Volume (mm^3) | Extrema Value | x | y | z | Label |
|  | 1 | 160 | 0.008859 | 58 | -53 | -21 | Right Fusiform Gyrus. |
|  | 2 | 160 | 0.009177 | 38 | 47 | -12 | Right Middle Frontal Gyrus |
|  | 3 | 160 | 0.008859 | -40 | 48 | -8 | Left Inferior Frontal Gyrus. |
|  | 4 | 160 | 0.008973 | -56 | -52 | 2 | Left Middle Temporal Gyrus |
|  | 5 | 160 | 0.00886 | -14 | 16 | 6 | Left Caudate Body |
|  | 6 | 160 | 0.009177 | 54 | -52 | 22 | Right Superior Temporal Gyrus |
|  | 7 | 160 | 0.009177 | 20 | 39 | 40 | Right Medial Frontal Gyrus |
|  | 8 | 152 | 0.009506 | 10 | 14 | 8 | Right Caudate Body |
|  | 9 | 64 | 0.008552 | -47 | -27 | 23 | Left Insula |
|  | 10 | 56 | 0.007556 | 18 | -74 | -38 | Right Cerebellum Crus II |
|  | 11 | 56 | 0.007556 | -30 | -14 | -24 | Left Parahippocampal Gyrus Hippocampus |
|  | 12 | 56 | 0.007556 | -32 | 28 | -12 | Left Inferior Frontal Gyrus |
|  | 13 | 56 | 0.007556 | -12 | 26 | -8 | Left Caudate Head |
|  | 14 | 56 | 0.007556 | 0 | -96 | -4 | Left Lingual Gyrus |
|  | 15 | 56 | 0.007556 | 0 | 66 | 8 | Left Medial Frontal Gyrus |
|  | 16 | 56 | 0.007556 | -56 | -54 | 20 | Left Superior Temporal Gyrus |
|  | 17 | 56 | 0.007556 | 66 | -4 | 24 | Right Precentral Gyrus |
| **Adults: ADHD < TD** | 1 | 160 | 0.008618 | -15 | -94 | 17 | Left Superior Occipital Gyrus |
|  | 2 | 160 | 0.008618 | 10 | -91 | 27 | Right Cuneus |
|  | 3 | 152 | 0.009739 | 6 | 18 | -14 | Anterior Cingulate Gyrus |
|  | 4 | 152 | 0.008508 | -18 | -40 | 22 | Left Caudate Tail |
|  | 5 | 152 | 0.008405 | 18 | 6 | 22 | Right Caudate Body |
|  | 6 | 152 | 0.008508 | 16 | -32 | 42 | Right Cingulate Gyrus |
|  | 7 | 152 | 0.008404 | 18 | 2 | 44 | Right Cingulate Gyrus |
|  | 8 | 152 | 0.009739 | 48 | 18 | 44 | Right Middle Frontal Gyrus |
|  | 9 | 152 | 0.009739 | 8 | 24 | 64 | Right Supplementary Motor Area |
|  | 10 | 96 | 0.007415 | 23 | -33 | 6 | Right Hippocampus |
|  | 11 | 96 | 0.007415 | 57 | -40 | 25 | Right Supramarginal Gyrus |
|  | 12 | 80 | 0.007331 | 23 | 14 | -32 | Right Superior Temporal Gyrus |
|  | 13 | 80 | 0.007332 | 26 | 2 | 8 | Right Putamen |
|  | 14 | 80 | 0.007649 | 11 | -48 | 18 | Right Precuneus |
|  | 15 | 64 | 0.007649 | -3 | 42 | -32 | Left Orbitofrontal Cortex |
| **Children: Dyslexia < TD** | 1 | 536 | 0.011585 | -56 | 8 | -16 | Left Superior Temporal Gyrus |
|  | 2 | 456 | 0.010942 | -48 | -46 | 28 | Left Inferior Parietal Lobule |
|  | 3 | 160 | 0.009758 | -5 | -20 | 5 | Left Thalamus Medial Dorsal Nucleus |
|  | 4 | 96 | 0.007666 | 40 | -86 | -14 | Right Inferior Occipital Lobe |
|  | 5 | 96 | 0.007665 | -60 | 6 | 20 | Left Precentral Gyrus |
|  | 6 | 96 | 0.008211 | -20 | -74 | 20 | Left Cuneus |
|  | 7 | 96 | 0.007665 | -7 | 60 | 27 | Left Medial Superior Frontal Gyrus |
|  | 8 | 96 | 0.008211 | -28 | 18 | 42 | Left Middle Frontal Gyrus |
|  | 9 | 88 | 0.007975 | -10 | -94 | 0 | Right Calcarine |
|  | 10 | 80 | 0.007913 | 6 | 53 | -22 | Right Medial Frontal Gyrus |
|  | 11 | 80 | 0.007913 | 13 | -36 | 0 | Right Parahippocampal Gyrus |
|  | 12 | 80 | 0.007654 | -34 | 18 | 10 | Left Insula |
|  | 13 | 80 | 0.007649 | -32 | 32 | 28 | Left Middle Frontal Gyrus |
|  | 14 | 80 | 0.007724 | 34 | -18 | 50 | Right Precentral Gyrus |
|  | 15 | 64 | 0.007855 | 36 | -18 | 42 | Right Postcentral Gyrus |
|  | 16 | 64 | 0.006733 | -54 | -50 | 48 | Left Inferior Parietal Lobe |
|  | 17 | 56 | 0.00817 | -6 | 52 | -22 | Left Medial Frontal Gyrus |
|  | 18 | 56 | 0.008173 | -20 | -60 | 8 | Left Calcarine |
| Children: ADHD < TD | 1 | 216 | 0.010117 | 10 | 30 | -20 | Right Medial Frontal Gyrus |
|  | 2 | 160 | 0.009492 | 24 | -6 | 4 | Right Lateral Globus Pallidus |
|  | 3 | 96 | 0.009446 | 34 | -12 | 62 | Right Precentral Gyrus |
|  | 4 | 80 | 0.009673 | -16 | -48 | -44 | Left Cerebellum lobule IX |
|  | 5 | 80 | 0.009673 | 21 | -86 | 2 | Right Calcarine Cortex |
|  | 6 | 80 | 0.009673 | 10 | 12 | 6 | Right Caudate Body |
|  | 7 | 80 | 0.009673 | -8 | 18 | 9 | Left Caudate Body |
|  | 8 | 80 | 0.009673 | -6 | -98 | 12 | Left Superior Occipital Gyrus |
|  | 9 | 80 | 0.008563 | -26 | -23 | 68 | Left Precentral Gyrus |
|  | 10 | 64 | 0.008991 | 17 | -47 | -45 | Right Cerebellum lobule IX |
|  | 11 | 64 | 0.008007 | -33 | -27 | -29 | Left Fusiform Gyrus |
|  | 12 | 64 | 0.008052 | 18 | 28 | -18 | Right Orbitofrontal Gyrus |
|  | 13 | 64 | 0.008484 | -7 | -9 | 47 | Left Cingulate Gyrus |
|  | 14 | 64 | 0.008007 | -19 | -37 | 63 | Left Postcentral Gyrus |
